# Selective Laser Melting of Pre-Alloyed NiTi Powder: Single-Track Study and FE Modeling with Heat Source Calibration

**DOI:** 10.3390/ma14237486

**Published:** 2021-12-06

**Authors:** Stanislav V. Chernyshikhin, Denis G. Firsov, Igor V. Shishkovsky

**Affiliations:** Center for Design, Manufacturing and Materials, Skolkovo Institute of Science and Technology, 121205 Moscow, Russia; D.Firsov@skoltech.ru

**Keywords:** SLM, nickel–titanium, shape-memory alloys (SMAs), single track, process parameter optimization

## Abstract

Unique functional properties such as the low stiffness, superelasticity, and biocompatibility of nickel–titanium shape-memory alloys provide many applications for such materials. Selective laser melting of NiTi enables low-cost customization of devices and the manufacturing of highly complex geometries without subsequent machining. However, the technology requires optimization of process parameters in order to guarantee high mass density and to avoid deterioration of functional properties. In this work, the melt pool geometry, surface morphology, formation mode, and thermal behavior were studied. Multiple combinations of laser power and scanning speed were used for single-track preparation from pre-alloyed NiTi powder on a nitinol substrate. The experimental results show the influence of laser power and scanning speed on the depth, width, and depth-to-width aspect ratio. Additionally, a transient 3D FE model was employed to predict thermal behavior in the melt pool for different regimes. In this paper, the coefficients for a volumetric double-ellipsoid heat source were calibrated with bound optimization by a quadratic approximation algorithm, the design of experiments technique, and experimentally obtained data. The results of the simulation reveal the necessary conditions of transition from conduction to keyhole mode welding. Finally, by combining experimental and FE modeling results, the optimal SLM process parameters were evaluated as P = 77 W, V = 400 mm/s, h = 70 μm, and t = 50 μm, without printing of 3D samples.

## 1. Introduction

Shape-memory alloys (SMAs) are a type of smart material that have additional properties, such as the shape-memory (SM) effect or superelasticity (SE) behavior. SMAs are capable of returning to their original shape due to reverse martensite phase transformation, which can be induced by temperature or stress. Nickel–titanium (nitinol) intermetallic alloys with equiatomic composition are among the most applicable SMAs on the market. Among many applications—such as actuators, fittings, valves, etc. [1]—medical devices and implants are attracting increased attention [2]. The behavior of the SMA near ambient temperature is close to that of hard tissues such as human bone or tendons. High mechanical strength and relatively low modulus of elasticity are essential characteristics of the NiTi alloy as a biomedical material [3]. These properties make this SMA an excellent material for various implants, such as dental implants, joints, spine fixators, etc. [4].

Nitinol has a high recoverable deformation value, making it more attractive in the manufacturing process. However, machining NiTi alloys is challenging due to their high-stress hardening [5], unconventional stress–strain characteristics, and formation of burrs [6]. These limitations restrict the manufacturing of nitinol in the form of rods and plates [7], which require additional manufacturing steps such as cutting [8], welding [9], or drilling [10]. Conventional powder metallurgy and casting are also complicated due to the absorption of impurities [11], the formation of metastable intermetallic phases [7], and inhomogeneous mixing of the liquid phase [12]. As a result, attempts have been made to manufacture this material using different approaches, such as additive manufacturing (AM).

AM is recognized as a new trend in the production of complex geometric parts [13]. The AM approach includes preparing a 3D model of an object using computer-aided design (CAD), slicing the model into monolayers, followed by G-code synthesis. Selective laser melting (SLM) (also known as laser powder bed fusion) is an AM technology suitable for manufacturing metal parts with complex geometries with great precision. During the SLM process, the formation of the powder bed on top of the substrate or previous layer is followed by scanning of the layer, with a complete melting of the scanned regions. This process is repeated until the whole 3D part is consolidated in a layer-by-layer fashion. The ability to manufacture almost any geometric shape allows for lightweight constructions, topologically optimized parts, structural objects, patient-specific implants, etc. Furthermore, the combination of AM and SMA gives rise to ’4D printing’, involving consideration of altering the shape or properties over time what gives even new applications of nitinol [14,15]. However, despite all of the advantages of the AM approach, SLM is a complex physicochemical metallurgy process. For successful consolidation, fine-tuning of the process is required due to numerous possible defects, such as stress-induced cracking, residual porosity, lack of fusion, keyhole porosity, or deviation from prescribed dimensions [16].

Different research groups have reported dense NiTi parts produced via SLM under different printing conditions, such as laser power, preheating temperature of the substrate, powder fraction, size of the laser spot, etc. [16,17,18,19,20,21,22]. In this regard, it can be concluded that the window of possible process conditions could be shifted with different SLM equipment, raw powders, laser properties, and scanning strategies. It is necessary to develop a suitable approach for optimizing printing parameters. The multi-iteration optimization approach based on the printing of volumetric samples is straightforward and affordable for widespread and inexpensive materials. However, the above approach is not applicable if it is necessary to obtain the optimal 3D printing conditions for a limited time and a small amount of powder due to possible high cost of material. On the other hand, a minimum amount of powder is required in order to obtain tracks and study the geometry of the melt pool. To date, single-track studies have been performed on materials such as cp-Ti [23], IN625 [24], SS316L [25], Ti_6_Al_4_V [26], AlSi_10_Mg [27], etc. However, comprehensive research dedicated to nitinol has not been reported, despite the high application potential of additively manufactured NiTi parts.

To predict the temperature field during the SLM process, finite element (FE) modeling is considered a powerful tool. An FE model of the SLM process considering metal gasification pressure and heat dissipation was proposed in [28]. A model for predicting the penetration depth of the bead into the substrate, and a proposal for a new heat source with consideration of laser reflections, were reported in [29]. An FE model for SLM, considering the powder layer’s shrinkage during the powder-to-solid transition, was established in [30]. Despite these advances in the numerical modeling of the SLM process, research on the thermal behavior and melt pool formation of nickel–titanium alloy has been presented only for other manufacturing technologies. The authors of [31] studied the melt pool formation in the directed-energy deposition (DED) of pre-alloyed nitinol powder, and developed an FE model to investigate the temperature gradient and solidification rate during cladding. The authors of [32] demonstrated wire-arc-based additive manufacturing of NiTi thin-wall structures with laser marking treatment to decrease the melt pool instability, and simulated temperature distributions on the titanium substrate during the process. In [33], an FE model of laser welding was used for two nitinol sheets, using Goldak’s heat source and a neural network to select the coefficients for the heat source. However, the presence of the powder bed makes the SLM process different from the laser welding, and greatly affects the results of the modeling.

In the present study, single tracks of NiTi powder were manufactured under a broad range of process parameters involving most of the reported combinations in the literature [16,17,18,19,20,21,22]. The experiment was performed with 24 combinations of laser power and scanning speed. To investigate the thermal behavior of nitinol powder during the SLM process, a transient 3D FE model based on solutions of heat transfer equations was established. The volumetric laser heat source was calibrated using our own experimentally obtained data. This work aims to study the correlation between the melt pool dimensions, thermal behavior, and process parameters—i.e., laser power and scanning speed—in order to evaluate the optimal regimes for SLM of NiTi powder.

## 2. Materials and Methods

For the experiments, a pre-alloyed NiTi powder produced by JSC Polema (Tula, Russia) was used. The atomization was carried out with an electrode induction-melting gas atomization (EIGA) technique. The powder was characterized using a Vega 3 SEM (TESCAN, Brno, Czech Republic) with EDS analysis. Granulometric analysis of the particles was carried out using an Analysette 22 laser particle sizer (Fritsch, Idar-Oberstein, Germany). The apparent density of the powder, according to the specifications, was 3.53 g/cm^3^.

The experiment was performed on an AddSol D50 (Additive Solutions, Moscow, Russia) SLM machine equipped with a CW ytterbium fiber laser (wavelength of 1070 nm). The laser beam profile yielded Gaussian power density distribution (TEM00), with a spot size diameter of 55 μm and a maximum laser power of 400 W. The chamber was filled with argon to prevent nitinol samples from assimilating hydrogen, nitrogen, and oxygen; the oxygen content in the chamber was less than 0.01%. An in-house NiTi substrate was built from a commercially available sheet with a thickness of 4 mm. The substrate was lowered by 50 μm; without changing its position, it was filled with powder via a recoating cycle with extra powder feeding in order to ensure complete coverage of the substrate with powder, as well as homogeneous powder distribution on the surface. The obtained powder layer was scanned with the laser according to the executive file prepared with the Glicer build processor (version 2.0.1, Moscow, Russia), resulting in single tracks. The printing parameters and linear energy density reported in the literature are shown in Table 1; the set of parameters chosen for this study is presented in Table 2.

After the SLM procedure, the single tracks with the substrate were cut with a GX-320L electrical discharge machine (CHMER EDM, Taichung, China) to obtain cross-sections of printed single tracks. For each combination of parameters, four unique locations of every single track were examined. Afterward, the samples were mounted in resin and polished using TechPress and MetPrep (Allied, East Rancho Dominguez, CA, USA) equipment. To visualize the contrast between the substrate and single tracks, the samples were slightly etched with an acidic solution of HF + HNO_3_ + H_2_O in the following proportions: 5% + 15% + 80%, respectively. All samples were studied with an Axioscope A1 optical microscope (Zeiss, Oberkochen, Germany) at the next stage.

## 3. FE Modeling of the Melt Pool

For the FE model, COMSOL Multiphysics (version 5.6, Burlington, MA, USA) software was used to calculate temperature fields. The model is based on solving heat transfer equations with the volumetric laser heat source. The model considers the following steps occurring during SLM: scanning of the powder layer with a moving laser beam (represented by volumetric heat source); absorption of laser radiation by a powder medium; melting of solid material; and heat transfer between the material, the substrates, and the surrounding atmosphere. The model has the following assumptions:

- Fluid dynamics are not taken into account;
- The evaporation is not taken into account;
- The powder bed is homogeneous, continuous, and flat;
- Isotropic heat conduction.

### 3.1. Governing Equations

The simulation is based on the governing equation with the following terms:

(1) $$\rho \left(T\right)C_{p}\left(T\right)\frac{\partial T}{\partial t}=\frac{\partial }{\partial x}\left(k\left(T\right)\frac{\partial T}{\partial x}\right)+\frac{\partial }{\partial y}\left(k\left(T\right)\frac{\partial T}{\partial y}\right)+\frac{\partial }{\partial z}\left(k\left(T\right)\frac{\partial T}{\partial z}\right)+Q$$

where $\rho \left(T\right)$ is the mass density, $C_{p}\left(T\right)$ is the specific heat capacity, $T\left(x,y,z,t\right)$ is the temperature field, $k\left(T\right)$ is the thermal conductivity, and Q is the heat absorbed during the laser’s interaction with the powder bed. Expression (1) is a 3D transient heat conduction equation in a Cartesian coordinate system derived from Fourier’s law. The initial conditions represented by the constant temperature field $T_{0}$ at the time t=0 are as follows:

(2) $$T\left(x,y,z,0\right)=T_{0}\left(x,y,z\right)\;\;\left(x,y,z\right)\epsilon \Omega$$

where $T_{0}\left(x,y,z\right)$ is the ambient temperature, assumed as 293 K. The model considers heat loss due to radiation and convection. The boundary conditions are represented by Equation (3) as the heat flux at the top surface:

(3) $$-k\left(T\right)\frac{\partial T}{\partial n}=h_{c}\left(T-T_{0}\right)+\varepsilon \sigma \left(T^{4}-T_{0}^{4}\right)$$

where n is the normal vector to the top surface, $h_{c}$ is the convection heat transfer coefficient, ε is the emissivity of the material, and σ is the the Stefan–Boltzmann constant.

### 3.2. Volumetric Heat Source

In the present model, the heat input was represented by the Goldak heat source described in [34]. The following equations describe the heat fluxes for the front and rear parts of the heat source:

(4) $$q_{f}\left(x,y,z,t\right)=\frac{6\sqrt{3}f_{f}\mathrm{AQ}}{{\mathrm{abc}}_{f}\pi \sqrt{\pi }}\exp(-\frac{3{\left(x-x_{0}-\mathrm{Vt}\right)}^{2}}{c_{f}{}^{2}}-\frac{3{\left(y-y_{0}\right)}^{2}}{b^{2}}-\frac{3{\left(z-z_{0}\right)}^{2}}{a^{2}})$$

(5) $$q_{r}\left(x,y,z,t\right)=\frac{6\sqrt{3}f_{r}\mathrm{AQ}}{{\mathrm{abc}}_{r}\pi \sqrt{\pi }}\exp(-\frac{3{\left(x-x_{0}-\mathrm{Vt}\right)}^{2}}{c_{r}{}^{2}}-\frac{3{\left(y-y_{0}\right)}^{2}}{b^{2}}-\frac{3{\left(z-z_{0}\right)}^{2}}{a^{2}})$$

**Figure 1:**
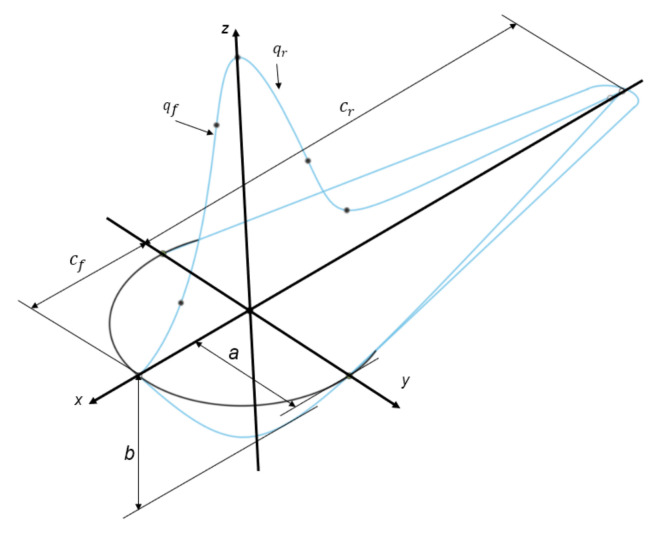
Scheme of the volumetric laser heat source.

The heat source equation includes several coefficients, which must be selected empirically or determined experimentally. The coefficients $a,\;b,{\;c}_{r},{\;c}_{f}$ define the geometry of the volumetric heat source as shown in Figure 1. The coefficients $f_{f}$ and $f_{r}$ are fractions of deposited heat for the front and rear parts of the laser beam, respectively, such that $f_{f}+f_{r}=2$. The motion of the heat source along the x-axis is represented by $\left(x-x_{0}-\mathrm{Vt}\right),$ where $x_{0}$ represents the initial coordinates of the laser’s influence. For convenience, the simulation time period was set to $\frac{0.45L}{V}$, where L is the length of the powder bed (1 mm) and V is the scanning speed of the laser beam (Table 2). The timestep was set to $\frac{1}{50}$ of the time period.

### 3.3. Thermal Properties

For an accurate simulation of the process, the values of the thermophysical parameters and their temperature dependences are of great significance. The model assumes the following states: initial loose powder, a liquid state in the melt pool, and a solid state after cooling. The values for both liquid- and solid-state mass density are measured experimentally in [35], while the temperature dependences of heat capacity and thermal conductivity for the solid state are measured experimentally in [36]; for the liquid state, there is no information available. All thermal physical properties of NiTi utilized in the model are presented in Table 3.

The powder bed is considered to be a porous medium consisting of solid particles and gas-filled pores. As a result, the effective thermal conductivity depends to a great extent on the powder properties (powder sphericity, size distribution, etc.) and gas properties (thermal conductivity of gas). The effective thermal conductivity for spherical powder in a gas atmosphere is defined by the empirical Equation (6), taken from [38]. The temperature dependence of mass density is given in Equation (7).

(6) $$k_{p}\left(T\right)=k_{s}\left(T\right){\left(1-\varphi \right)}^{n}$$

(7) $$\rho \left(T\right)=\left\{\begin{array}{cc} \rho_{p} & T\leq T_{s}\\ \frac{T-T_{s}}{T_{l}-T_{s}}\left(\rho_{l}-\rho_{p}\right)+\rho_{p} & T_{l}\leq T\leq T_{s}\\ \rho_{l} & T\geq T_{l} \end{array}\right.$$

(8) $$\varphi =\frac{\rho_{s}-\rho_{p}}{\rho_{s}}$$

where φ is the degree of density of the loose powder in relation to the solid state, defined by Equation (8), while $\rho_{p}$ is set to 3.53 g/cm^3^ according to the powder specification. The change in density with temperature was not considered due to the weak dependence. The differences in mass density for the liquid and solid states were taken into account.

The heat capacity temperature dependence involves singularity due to the latent heat of fusion $L_{1\to 2}\frac{\partial \alpha }{\partial T}$ during the phase transition. Temperature dependence for specific heat capacity is given by the following equations [39]:

(9) $$C_{p}\left(T\right)=\left\{\begin{array}{cc} C_{p}\left(T\right) & T\leq T_{s}\\ \theta_{1}C_{p1}+\theta_{2}C_{p2}+L_{1\to 2}\frac{\partial \alpha }{\partial T} & T_{l}\leq T\leq T_{s}\\ C_{p2} & T\geq T_{l} \end{array}\right.$$

(10) $$\theta_{1}+\theta_{2}=1$$

**Figure 2:**
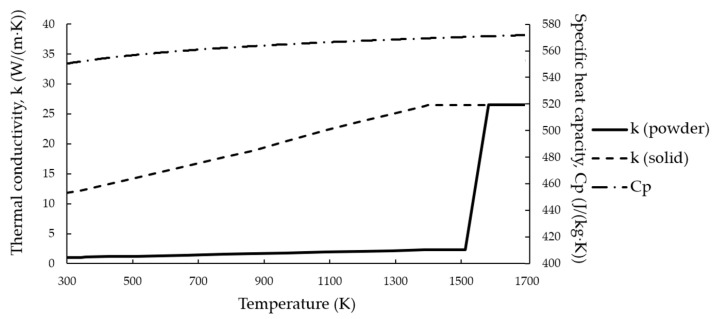
Temperature dependences on thermal conductivity and specific heat capacity.

The temperature dependences of calculated effective thermal conductivity, thermal conductivity of solid NiTi, and specific heat capacity [36] are shown in Figure 2, in the range from ambient temperature up to the melting point. Due to the lack of experimental data on thermophysical properties for both properties, the values measured at the highest temperature were used for the liquid state as constants.

### 3.4. Geometry, Mesh, and Heat Source Calibration

**Figure 3:**
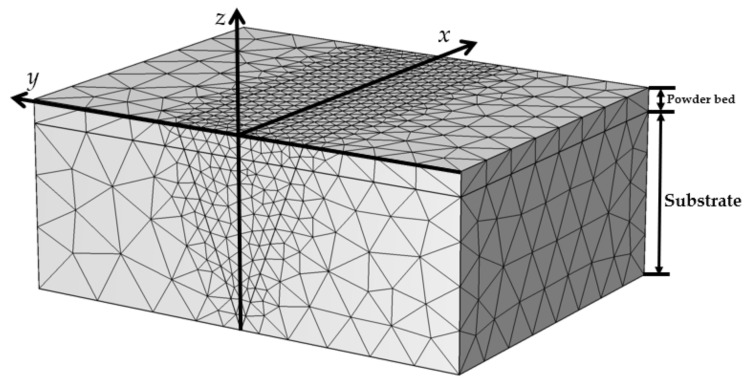
The geometry of the powder bed and substrate, with a finer mesh along the direction of the laser’s movement.

The substrate was represented as a block with 350 µm height, 1 mm width, and 1 mm length. The NiTi powder bed was represented as an upper layer with the same dimensions, except that the block height was 50 µm in accordance with the experimental setup. The mesh was built with 6980 tetrahedral elements, yielding an element size distribution from 10 µm to 100 µm. For the volume subjected to laser influence, mesh convergence analysis was carried out in order to find the optimal size of elements; for the remaining geometry, the mesh was coarser in order to save computational time. The overall geometry and mesh are shown in Figure 3.

The Goldak heat source coefficients depend on the material, process parameters, and laser source parameters. The coefficients a,b were considered to be dependent on laser power and scanning speed, and were found using the Bound Optimization BY Quadratic Approximation (BOBYQUA) algorithm [40]. BOBYQUA is a derivative-free optimization algorithm for the solving of bound-constrained problems. The trust region for both coefficients was $\left(10^{-6};400\cdot 10^{-6}\right)$, covering the whole thickness of the domain. The objective function is represented by the following equation:

(11) $$f\left(T_{1},T_{2}\right)={\left(T_{1}-T_{m}\right)}^{2}+{\left(T_{2}-T_{m}\right)}^{2}\to 0$$

where $T_{1}$ and $T_{2}$ are the calculated temperatures at the edge and bottom of the melt pool, respectively. Additionally, empirical models $a=f\left(P,V\right);b=f\left(P,V\right)$ were established using the statistical design of experiment (DOE) techniques presented in Equation (12). The custom response surface design was created using P and V as continuous factors within one block. The analysis showed the statistical significance of both factors. The response surface regression models were built including quadratic and interaction terms.

(12) $$\left\{\begin{array}{c} a=10^{-6}\cdot \left(148.8-2.493\;P\;+0.1824\;V\;+0.00865{\;P}^{2}-0.000294\;P\cdot V\right)\\ b=10^{-6}\cdot \left(35.3+3.32\;P\;-0.172\;V\;-0.00024{\;P}^{2}-0.00383\;P\cdot V\right) \end{array}\right.$$

The coefficients $c_{f},c_{r}$ were considered as primarily material- and laser-source-dependent; therefore, they were set to the constants $r_{b}$ and $4r_{b}$, respectively, according to the methodology described in [41,42]. The quadrant factors were set to $f_{f}=0.4,{\;f}_{r}=1.6$ as described by the authors of the original model [34] and, additionally, can be calculated by Equation (13), as derived and confirmed in [39]:

(13) $$f_{f}=2\frac{c_{f}}{c_{f}+c_{r}}=2-f_{r}$$

## 4. Results and Discussion

**Figure 4:**
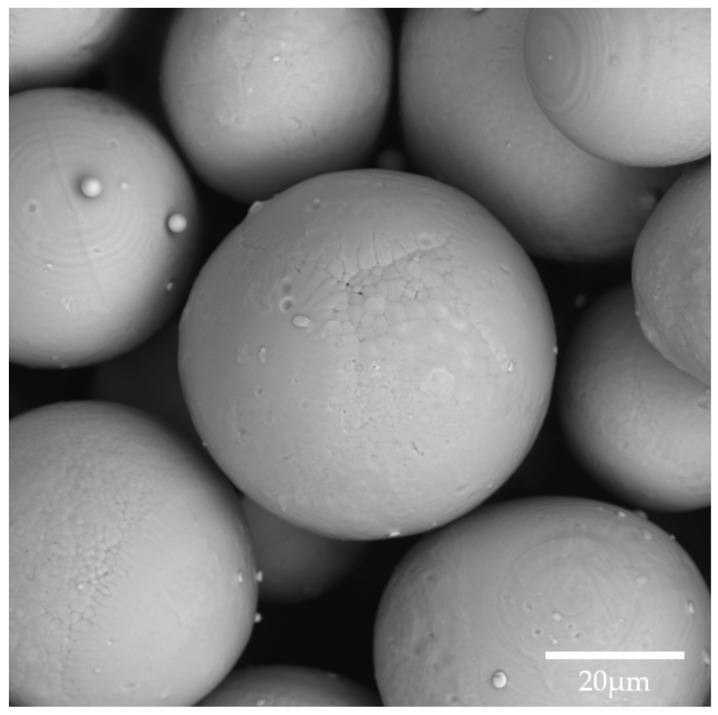
High-magnification SEM image of nitinol powder.

**Figure 5:**
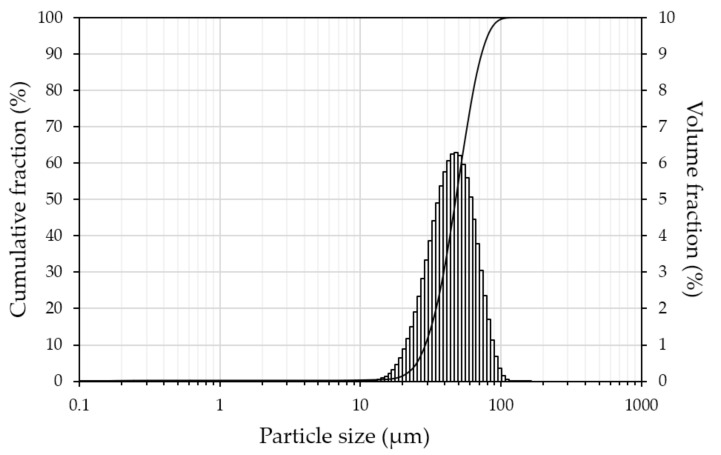
Particle size distribution of nitinol powder.

The composition of pre-alloyed nitinol powder was Ni 55.59 wt.% and Ti 44.42 wt.%. Figure 4 shows a high-magnification SEM image of the powder, revealing the micro-dendritic structure on the surface of the spherical particles inherent to the EIGA process. NiTi powder was represented by a 50 µm fraction with unimodal normal distribution, as shown in Figure 5. The percentiles of equivalent diameters were d_10_ = 28.2 µm, d_50_ = 46.4 µm, and d_90_ = 70.9 µm.

Investigation of the optimal combination of process parameters is crucial for manufacturing high-quality parts using SLM. Linear energy density $E_{l}$, as given by Equation (14), was used for the evaluation of energy input during a single laser scan. Volumetric energy density $E_{v}$, as given by Equation (15), was considered when the whole part is printing in a track-by-track and layer-by-layer fashion.

(14) $$E_{l}=\frac{P}{V}$$

(15) $$E_{v}=\frac{P}{\mathrm{Vht}}$$

where P is the laser power, V is the scanning speed, h is the hatch spacing, and t is the layer thickness.

**Figure 6:**
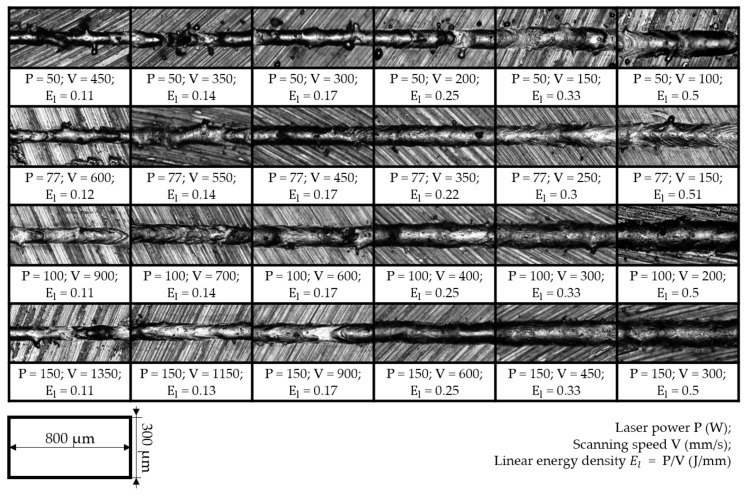
Micrographs of the single tracks’ top view.

In this study, the dependence of the melt pool dimensions for different $E_{l}$ levels were observed using various combinations of laser powers and scanning speeds. Top-view micrographs of the single tracks are presented in Figure 6. All images presented on the same scale clearly show that with a decrease in scanning speed, the width of the single tracks increased. Such analysis provides valuable information on the single tracks’ morphology and width.

The single tracks made with laser powers from 77 W to 150 W and an $E_{l}$ greater than 0.17 showed the most stable and continuous weld beads. The single track deposits obtained with a laser power of 50 W demonstrated humping behavior, and some unmelted powder particles attached to the beads could also be observed. In the case of higher speeds for all levels of laser power, the single tracks’ morphology resulted in wavy cylinders due to the Plateau–Rayleigh capillary instability phenomenon, as shown in [29]. With a further increase in scanning speed, the single track beads become discontinuous, commonly known as a balling effect [43]; however, in the chosen range of scanning speeds, such defects were not observed. Additionally, the surface roughness of the final part is greatly affected by the morphology of the single tracks. With an increase in linear energy density, the growth of the above-surface layer was observed. The excessive height of the beads complicates the formation of a homogeneous powder bed at the subsequent layers; as a result, tracks with a large above-surface layer will be remelted many times, increasing the curvature of the surface. In [44], the authors showed that both discontinuity and overheating of single tracks led to an increase in the surface roughness of the final parts.

With increasing laser power, an increase in the width of the single tracks was observed for the same levels of linear energy density. The smallest width of 58 μm was observed at P = 50 W and V = 450 mm/s, which is close to the laser beam diameter. The regimes with the highest $E_{l}$ level of 0.5 J/mm resulted in a significant expansion in the melt pool dimensions, up to three times the laser spot diameter. Measurements of the single beads’ width are essential before printing of 3D objects, in order to avoid multiple remelting of the same powder volume and guarantee the necessary overlap between adjacent tracks. Such analysis was presented for NiTi intermetallic powder in [17,37,45], where Realizer SLM 100 (SLM Solutions, Lübeck, Germany), Aconity3D Midi (Aconity3D, Herzogenrath, Germany), and Phenix PXM (now 3D Systems, Rock Hill, SC, USA) installations were used, respectively, all with a laser spot size of 80 μm. The reported results of the single tracks’ width measurements show the same tendencies; however, the values are higher than those obtained in this work. In [45], the width of the melt pool was described with a regression model as a function of laser power and scanning speed. The coefficients in regression Equation (16) are in good agreement with the present study, with a 2–5% difference—except for the laser power factor which is reduced by 23%. The installations used for the experiments all had ytterbium fiber lasers that yielded a Gaussian power density distribution (TEM00) and wavelength of 1070 nm. The intermetallic nitinol powders had the same particle size distribution of 25–75 μm. There was a significant difference in the laser spot diameter, which was 55 μm for the AddSol D50 installation (Section 2). To take into account the factor of laser spot size, Equation (17) was proposed. Results from the literature and experimentally obtained values correlate with the derived equation having a 5% error. Equation (17) can be used for the melt pool width calculation in order to evaluate the correct hatch spacing parameters (from Equation (15)), with consideration of ~20% overlapping between adjacent tracks.

(16) $$\omega =10^{-6}\left(0.5727\cdot P+32.743\right)V^{-0.432}$$

(17) $$\omega =10^{-6}\left(\left(0.0108\cdot r_{b}+0.1435\right)P+32.7\right)V^{-0.4}$$

**Figure 7:**
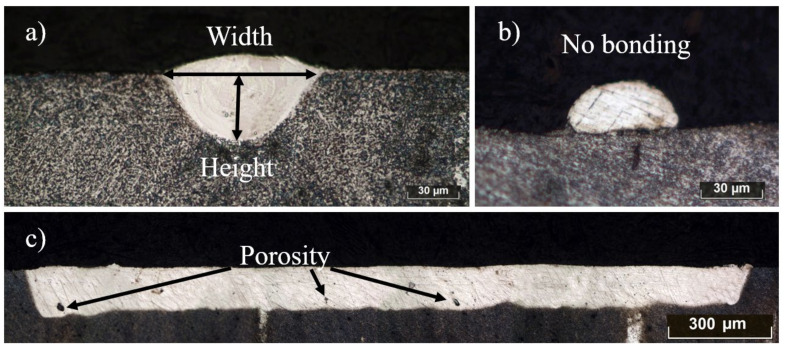
Cross-sections of NiTi single-track deposits: (**a**) common shape of the melt pool for the SLM process; (**b**) no bonding defect; (**c**) appearance of the pores during keyhole mode in the longitudinal cross-section.

A common single-track deposit cross-section is presented in Figure 7a. The primary geometric parameters of the melt pool are depth, width, and depth-to-width aspect ratio [26]. These parameters are strongly dependent on the process conditions and linear energy density. Insufficient penetration depth could result in poor metallurgical contact of the deposit with the substrate (the previous layer), or even lead to no contact at all (Figure 7b). Thus, the solidified bead could be displaced by the recoater blade during the formation of the next powder layer, leading to disturbance of the layer-by-layer consolidation. On the other hand, an excessive depth of the melt pool leads to the appearance of a keyhole defect. During the melting of the powder by a laser beam, intense convective flows occur in the melt pool due to the Marangoni effect: the surface tension gradient caused by a temperature gradient creates a flow from the center of the melt pool with higher temperature to its edges. Gas bubbles can be captured in the melt pool during the convection mixing of the liquid phase; the probability of their ejection decreases with an increase in the depth of the melt pool, since the lifetime of the liquid phase is in milliseconds. The micrograph of the longitudinal cross-section with pores caused by this phenomenon is presented in Figure 7c.

**Figure 8:**
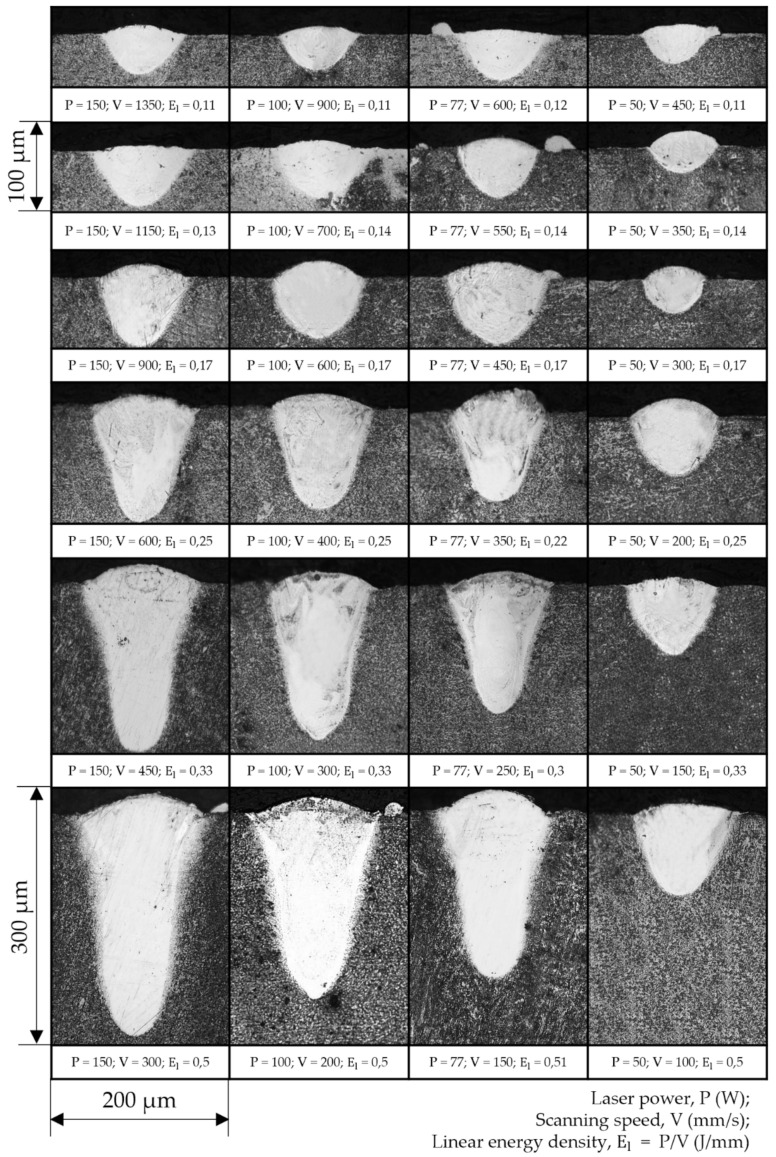
Cross-sections of single-track deposits for all combinations of parameters.

During laser welding, the optimal mode is the complete filling of the end surfaces with the liquid phase, i.e., the size of the melt pool is comparable with the size of the joint. On the contrary, for the SLM process, the appearance of a large melt pool is highly undesirable due to the appearance of a keyhole defect, as described above. With a change in the energy input, the depth and width change, along with the shape itself. From the presented micrographs of the single tracks’ cross-sections in Figure 8, the change in the geometry of the melt pool is visible. The bottom edges of the deposits are more spherical in cases of low values of linear energy density, and sharper with higher $E_{l}$ values. In the case of lower linear energy density, the surface tension forces of the liquid squeeze the surface due to the system’s tendency to minimize the surface energy, forming a cylindrical bead with moderate penetration into the substrate. On the other hand, the laser beam with high laser irradiation intensity and low scanning speed penetrates the material’s depth significantly, increasing the depth-to-width ratio and resulting in a narrow and deep melt pool.

**Figure 9:**
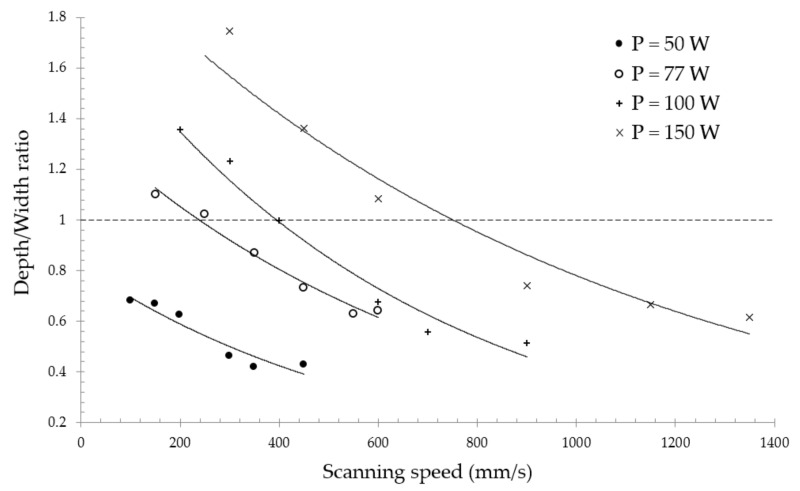
The depth-to-width aspect ratio for the experimentally obtained single tracks.

Figure 9 depicts the depth-to-width ratios for the experimentally obtained single tracks as a function of scanning speed for all laser power levels. The investigated regimes were divided into conduction mode and keyhole mode from the cross-sectional images and the aspect ratios. The combinations of process parameters with laser power of 150 W, 100 W, and 77 W, and scanning speed less than 600 mm/s, 300 mm/s, and 250 mm/s, respectively, were considered as keyhole mode regimes, with a depth-to-width ratio greater than 1; in the case of a lower aspect ratio, the melt pool formation occurs in a conductive mode. The authors of [25] studied the transition from conduction mode to keyhole mode, and proposed Equation (18) for the evaluation of the laser speed threshold below which keyhole mode appears. However, the experimentally obtained threshold of the scanning speed was higher than that calculated using the analytical expression.

(18) $$V=\frac{4\alpha }{r_{b}}{\left(\frac{\pi^{\frac{3}{2}}{\mathrm{kT}}_{b}r_{b}}{\mathrm{AP}}\right)}^{-2}$$

**Figure 10:**
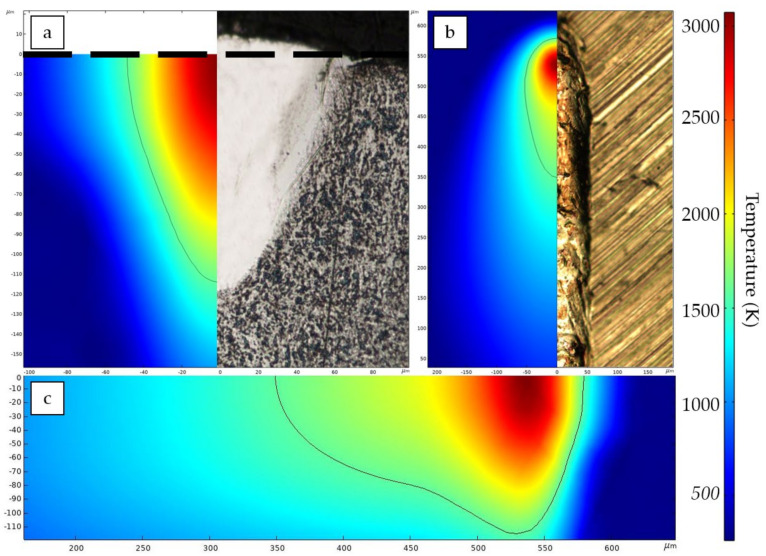
Calculated temperature field for the P = 100 W and V = 400 mm/s: (**a**) transverse cross-section at the center of the laser beam; (**b**) top view; (**c**) longitudinal cross-section. The solid line represents the fusion zone boundary.

**Figure 11:**
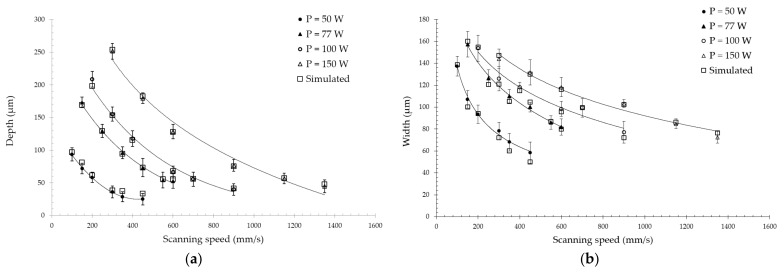
Experimental and simulated results on the (**a**) depth and (**b**) width of the melt pool.

A flexible double-ellipsoidal heat source (Section 3.2) was chosen for the obtained experimental data, since the cross-sections of the single tracks had both sharp and spherical shapes. The process conditions of the experiment described earlier were used for the FE simulations. The volumetric heat source was calibrated with the obtained experimental data, and the calculated temperature field for P = 100 W and V = 400 mm/s is demonstrated in Figure 10. The transverse cross-section (Figure 10a) and top view (Figure 10b) were compared with experimental images obtained from single tracks. The longitudinal cross-section (Figure 10c) reveals the difference in the melt pool depth in the front and rear parts. Compared to the experimentally measured single tracks’ width and depth, the overall simulation results are shown in Figure 11. Good agreement of the results confirms the effectiveness of the calibration method used in this study. Still, the implementation of the proposed model requires experimental data for calibration, and the result of calibration depends on many conditions, such as laser power density distribution, laser spot size, chemical composition of the material, powder particle size distribution, etc. However, the proposed model has its advantages—simplicity in implementation, speed of calculations, fewer required thermal physical properties for the material, and high agreement with the experimental results. The author of [46] showed that an FE model based on isotropic thermal conductivity with a well-calibrated double-ellipsoid heat source provides a maximum deviation of 7.3% from the experimental data. Furthermore, Kollmannsberger et al. proposed improvements of the experiment-based FE models by introducing anisotropic conductivities to address the contribution of thermal convection in the melt pool discussed above.

**Figure 12:**
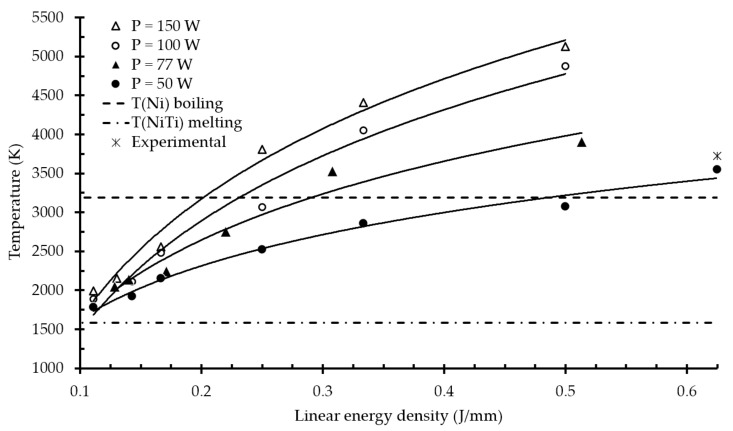
Peak temperature at the center of the laser beam for all combinations of parameters. The experimental result marked with an asterisk was taken from [49].

In Figure 12, the calculated peak temperatures in the melt pool are presented as a function of linear energy density. It was found that regimes with linear energy density levels of 0.25 J/mm, 0.31 J/mm, and 0.33 J/mm and laser power of 77 W, 100 W, and 150 W, respectively, had a maximum temperature at the center of the laser beam above the boiling point of nickel. In the lowest power case of 50 W, all temperatures were between the melting and boiling points. As far as the model does not take into account the heat dissipation due to evaporation, the values highly exceeding the boiling temperature (>4500 K) seemed to be overestimated. However, the maximum temperature value is proportional to the energy that will be spent on the formation of the gaseous phase and dissipated heat.

For experimental monitoring of temperature during SLM, a two-color high-temperature pyrometer is generally used [47,48]. The authors of [49] investigated the peak temperatures in the melt pool during the SLM process for NiTi powder. The reported measured value for the laser power of 50 W and scanning speed of 80 mm/s was 3723 K, as denoted by an asterisk in Figure 12. The peak temperature predicted by the developed FE model was 3558 K, with an error of less than 5%.

**Figure 13:**
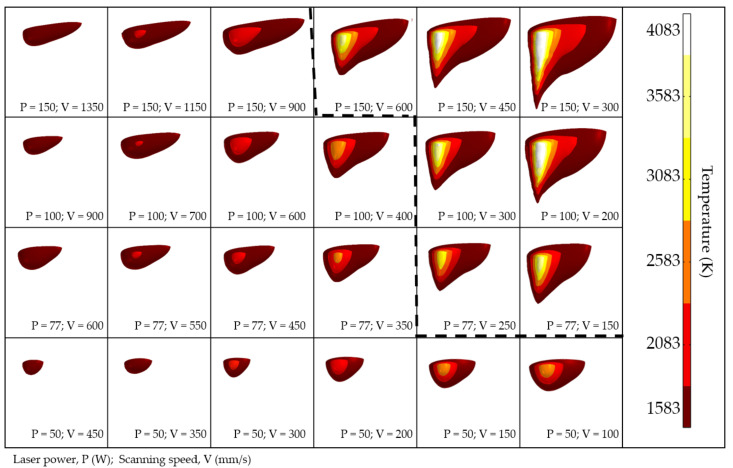
Evolution of the melt pool’s shape and temperature distribution with the process parameters. The dashed line divides the regimes into conductive (**left**) and keyhole (**right**) modes.

Calculated shapes of the melt pool are presented in Figure 13 as a combination of isothermal surfaces, where the lowest temperature is the NiTi liquidus. For clarity, the melt pool is cut along the plane of symmetry, and the temperature of the subsequent isosurfaces rises with a 500 K increment. The resulting melt pool shapes are divided into conductive and keyhole modes according to their experimentally measured depth-to-width aspect ratio. From the evolution of the melt pool shape and temperature field, it can be seen that a necessary condition for the appearance of keyhole mode is an increase in the temperature at the center of the laser’s influence, up to the boiling point. This phenomenon is associated with the fluid dynamics effects arising in the melt pool. The surface tension of metal tends to minimize the surface area of liquid metal, and the surface tension temperature dependence is inversely proportional for liquid metals. As discussed, due to the thermal gradients caused by non-uniform power density distribution on the surface, the fluid will transfer from the laser beam’s center to the edges of the melt pool. In addition, the gasification recoil force acts along the normal vector to the surface of the melt pool, pushing the liquid into the depth [50]. This force is proportional to the temperature and increases exponentially. As a result of the superposition of the stated forces, the morphology of the depression zone is formed. At high temperatures, the pressure force prevails over the Marangoni effect, causing the melt pool to be sharp and deep. The opposite is observed at lower temperatures, where the surface tension effect dominates, making the melt pool more spherical and wider [28].

Experimentally obtained single tracks for the laser power of 50 W have more attached particles (Figure 6) due to the less pronounced effect of denudation compared to higher laser powers. The calculated peak temperatures in the melt pool demonstrate the lowest increment with increasing linear energy density; additionally, the overall size of the melt pool for this case is significantly smaller, from which it follows that the lifetime of the liquid is shorter before it solidifies. Thus, the powder has a lower tendency to retrain into the melt pool; therefore, some powder attached to the single track can be observed. The authors of [51] showed that the mechanism of denudation zone formation during SLM for the high ambient pressure is connected with Bernoulli-effect-driven gas flow. During the vaporization of metal at the center of the laser’s influence, a metal jet appears. This vapor flow causes an inward ambient gas flow, entraining the closest particles into the melt pool.

In the production of parts via the SLM method, an insufficient energy input leads to defects—represented as closed pores with remaining powder—due to lack of fusion, which strongly impairs the mechanical performance of a part [37]. On the other hand, an excessive energy input will lead to the formation of spherical pores due to solidification of melt pool with captured gas bubbles [52]. In addition to the aforementioned defects, the following constraints must also be considered for the manufacturing of NiTi via SLM: Firstly, the increase in energy density input is undesirable due to the increase in pick-up of impurities during consolidation from the surrounding atmosphere [17]. It should be noted that despite the process being carried out in an airtight chamber filled with inert gas, the pick-up of oxygen, nitrogen, and carbon cannot be fully avoided. As a matter of fact, an increase in the content of impurities above 500 ppm is unacceptable for medical applications, according to the established ASTM F2063-05 standard. Secondly, the metal vapor jet in the melt pool results in nickel-depleted chemical composition, since the evaporation temperature of nickel (3186.15 K) is lower than that of titanium (3560.15 K) [53]. Consequently, the martensitic phase transition temperature is shifted to higher values, obstructing the manufacturing of pseudoplastic parts. Furthermore, crack generation has a higher tendency to appear in the case of higher temperature gradients, due to the relatively low thermal conductivity coefficient of nitinol. In [16], pre-heating up to 500 °C was shown to achieve more dense parts and prevent crack formation. The developed FE model can be used for predicting thermal behavior during SLM in order to accomplish the above-stated constraints. In such a way, combinations of laser power and scanning speed with peak temperature exceeding the boiling point (Figure 13, right) should be ignored so as to prevent Ni depletion. Among the remaining regimes, the ones with less input energy are preferred in order to minimize the pick-up of impurities and the tendency for crack generation. Additionally, single tracks should have homogenous and continuous morphology (Figure 6) along with a conductive-mode melt pool (Figure 8) in order to guarantee production of parts with the highest relative density. In this regard, the regime with laser power of 77 W and scanning speed of 450 mm/s is recommended for the manufacturing of 3D parts with a hatch distance of 70 μm (calculated from Equation (17), considering ~20% overlapping of adjacent tracks).

## 5. Conclusions

An experimental analysis of the melt pool geometry formed during the SLM of intermetallic nitinol powder was carried out in the present work. The geometric properties of the melt pool provide helpful information for the selection of the process parameters. Such an approach significantly reduces the time and cost of the optimization process. A numerical model with a double-ellipsoid volumetric heat source was established. The calibration of Goldak’s heat source coefficients based on a bound-constrained algorithm was carried out with experimentally obtained NiTi single tracks. Finally, the temperature fields and shapes of the melt pool were obtained for the combination of process parameters used in the experiment. Accordingly, the following main conclusions can be drawn from this study:

- It was shown based on the experimental investigation of the melt pool dimensions and morphology, along with FE modeling of the thermal field, that the optimal process parameters can be ascertained. In this study, an optimal regime of SLM for NiTi was found: P = 77 W, V = 400 mm/s, h = 70 μm, and t = 50 μm;
- It was demonstrated that the necessary condition of keyhole mode melting is exceeding the boiling point of the Ni. For laser powers of 77 W, 100 W, and 150 W the scanning speed thresholds are 150 mm/s, 300 mm/s, and 600 mm/s, respectively. For the laser power of 50 W, only conduction mode melting was observed in the studied scanning speed interval—from 450 mm/s to 100 mm/s. The appearance of the keyhole mode resulted in a deeper and sharper melt pool, with an experimentally measured depth-to-width ratio greater than 1;
- It was found that thermal behavior in the melt pool is highly affected by both laser power and scanning speed. The peak temperature is proportional to the linear energy density; however, for the same linear energy density levels, an increase in laser power leads to higher peak temperatures in the melt pool;
- A peak temperature of 3558 K in the melt pool was predicted using the developed model for a laser power of 50 W and scanning speed of 80 mm/s, which is in good agreement with the experimental data.

In future works, the developed heat transfer FE model will be coupled with solid mechanics for a comprehensive study of the thermal stresses induced during SLM of NiTi.

## Figures and Tables

**Table 1:** The parameter combinations for the printing of NiTi are reported in the literature.

Laser Power (W)	Scanning Speed (mm/s)	Hatch Distance (μm)	Layer Thickness (μm)	Reference
77	200	120	50	[17]
40	160	60	30	[18]
56	133	120	50	[19]
150	450	120	50	[20]
50	100,160	100	60	[16]
100	200	Lack of information	50	[21]
120	500	80	30	[22]
50–150	100–1350	Not applicable	50	This work

**Table 2:** The combinations of parameters used in the experiments.

Laser Power (W)	Scanning Speed (mm/s)					
50	450	350	300	200	150	100
77	600	550	450	350	250	150
100	900	700	600	400	300	200
150	1350	1150	900	600	450	300

**Table 3:** Thermal physical properties utilized in the FE model [35,37].

Property	Symbol	Value	Units
Density of powder	$\rho_{p}$	3.53	kg/m^3^
Density of liquid	$\rho_{l}$	6.05	kg/m^3^
Density of solid	$\rho_{s}$	6.45	kg/m^3^
Solidus temperature	$T_{s}$	1513	K
Liquidus temperature	$T_{l}$	1583	K
The boiling temperature of Ni	$T_{b}$	3033	K
Absorption coefficient	A	0.32	-
Melting latent heat	$L_{1\to 2}$	24 200	J/kg
Surface emissivity	ε	0.3	-
Stefan–Boltzmann constant	σ	5.67 × 10^−14^	W/(mm^2^·K)
Heat convection coefficient	$h_{c}$	20	W/m^2^

## Data Availability

The data presented in this study are available on request from the corresponding authors.
